# Members of the *Capsicum annuum* CaTrxh Family Respond to High Temperature and Exhibit Dynamic Hetero/Homo Interactions

**DOI:** 10.3390/ijms25031729

**Published:** 2024-01-31

**Authors:** Suji Hong, Sung Un Huh

**Affiliations:** Department of Biological Science, Kunsan National University, Gunsan 54150, Republic of Korea; hsj6550@gmail.com

**Keywords:** Thioredoxin (Trx), climate change, abiotic stress, *Capsicum annuum*

## Abstract

Climate change adversely affects the water and temperature conditions required for plant growth, leading to a decrease in yield. In high temperatures, oxidative stress causes cellular damage in plant cells, which is a negative factor for crop production. Thioredoxin (Trx) is a small redox protein containing a conserved WC(G/P)PC motif that catalyzes the exchange of disulfide bonds. It is known to play an important role in maintaining cellular redox homeostasis. Trx proteins are widely distributed across various subcellular locations, and they play a crucial role in responding to cellular stresses. In this study, seven *CaTrxh*-type genes present in pepper were identified and the *CaTrxh*-type family was classified into three subgroups. *CaTrxh* genes responded to heat stress. Moreover, subcellular locations of the CaTrxh family exhibited dynamic patterns in normal conditions, and we observed relocalizations in heat stress conditions. Each CaTrxh family protein member formed homo-/heteromeric protein complexes in BiFC assay. Unexpectedly, subgroup III CaTrxh9 and CaTrxh10 can recruit subgroup I and II CaTrxh proteins into the plasma membrane. Thus, the function of the CaTrxh-type family is expected to play a protective role in the cell in response to high-temperature stress via protein complex formations. CaTrxh may have potential applications in the development of crops with enhanced tolerance to oxidative stress.

## 1. Introduction

Crop production is decreasing due to global warming. Extreme temperature changes, droughts, and floods place great stress on plant growth [1]. In response to high temperatures, plants produce a variety of reactive oxygen species (ROS) as signaling molecules, and excessive ROS accumulation causes oxidative damage to organelles, proteins, and membranes [2]. To protect against oxidative damage, eukaryotic cells are equipped with a dynamic redox regulatory system [3]. In general, redox reactions affect the activity of various proteins that play important roles in metabolic processes.

Thioredoxin (Trx) is evolutionarily conserved in diverse organisms and plays important roles in many cellular processes involving redox regulation [4,5]. Trx is an about-12-kDa small protein with a redox-active disulfide motif (WCGPC) in a catalytic domain. Trx has a dithiol–disulfide oxidoreductase activity and functions as a reversible disulfide reductase that reduces target substrates [6]. During the reducing process, two cysteine residues at the ‘WCGPC’ active site are oxidized and inactivated. Maintaining basal levels of intracellular ROS is an essential process for cell differentiation and proliferation. Trx can associate with antioxidant enzymes to protect against intracellular oxidative damage via excess ROS accumulation in the cell. For example, *Arabidopsis thaliana Trx-h2*-overexpressing *Brassica napus* transgenic plants have shown enhanced antioxidant activity and have exhibited strong salt stress tolerance [7]. When Banana *MaTrx12* was heterologously overexpressed in *Trx*-deficient yeast cells, the mutant yeast exhibited improved viability against oxidative stress conditions [8]. Overexpression of *Drohophila Trx-2* has also been shown to prolong lifespan and increase resistance to oxidative stress [9]. These reports suggest that evolutionarily conserved Trx is a valuable genetic resource for the development of crops that can tolerate oxidative stress.

Plant Trx has evolved extensively, displaying a high degree of diversity compared to other organisms. The human has two TRX isoforms: TRX-1, found in the extracellular milieu, cytoplasm, and nucleus [10]; and TRX-2, which is located in the mitochondria and regulates mitochondrial metabolic activity [11]. In the Arabidopsis genome, a total of 41 Trx genes exist, which are further divided into 18 typical (WCGPC active site) and 23 atypical (XCXXC active site) Trx genes [12,13]. Plants higher than Arabidopsis are expected to have a more diverse Trx family. For example, 125 Trx were found in soybean (*Glycine max*) [14]. Nearly 86 GhTrx (40 typical and 46 atypical types) were identified in the cotton genome (*Gossypium hirsutum* L.) [15]. The Trx subfamily is also classified into seven types, namely, f, h, m, o, x, y, and z, based on sequence similarity, intracellular localization, and molecular function [13,16]. Among them, the largest family of Trx is the h-type, with 10 isoforms in Arabidopsis. AtTrxh-type is again divided into subtypes I (h1, h3, and h4), II (h2, h7, and h8), and III (h9, CxxS1, CxxS2, and CxxC2) [16]. In the rapeseed (*Brassica napus*) genome, it was confirmed that the Trxh subfamily consists of about 36 genes [7].

The Trxh-type function is still largely unknown. The AtTrxh-type proteins are expected to be present mainly in the cytoplasm, but most of the Trx proteins in crops are unknown. AtTrxh5 is associated with cytoplasmic oligomers of non-expressor PR1 (NPR1). NPR1 is translocated into the nucleus as a monomeric NPR1 and functions as a defense activator [17]. It has also been found that AtTrxh5 has a selective protein denitrosylation activity in plant immunity [18]. Interestingly, AtTrxh2 has been shown to exhibit an RNA chaperone-like activity under cold stress conditions and can distribute mRNA from the nucleus to the cytoplasm [19]. Recently, tomato (*Solanum lycopersicum*) *SlTrxh*-overexpressing tobacco plants have shown enhanced oxidative stress tolerance under excess nitrate stress, and SITrxh protein is associated with tomato peroxiredoxin (SlPrx) protein [20]. It can be expected that the functions of the subfamily belonging to the Trxh-type will be diverse. Identification of the Trxh-type in crops has not yet been [21] performed.

Pepper (*Capsicum annuum*), a member of the Solanaceae family, stands as one of the most crucial vegetable crops cultivated worldwide. Its versatility is showcased through its various uses as a spice, a medicinal ingredient, and as a staple in foods such as kimchi [21,22]. As a thermophilic vegetable, peppers contribute to the global market [23]. In this study, the *CaTrxh*-type subfamily identified in the hot pepper genome was analyzed. A total of seven *Trxh*-types were found, and we performed analysis of the CaTrxh protein sequence, gene expression, and subcellular localization. As a result, CaTrxh-type was highly conserved in the Solanaceae family of vegetables, including potato, tomato, and tobacco. The *CaTrxh* genes exhibited a significant response under heat stress conditions. The subcellular localizations of CaTrxh-type proteins have been observed in various intracellular locations. This diversification may imply complex biological functions of the CaTrxh-type family. In addition, CaTrxh protein members specifically formed protein complexes via homo-/heteromeric associations without the redox-active disulfide motif. It is expected that the characterization of the Trxh subfamily will enable the development of crops with strong antioxidant and chaperon-like functions that can respond to climate change.

## 2. Results

### 2.1. Genome-Wide Identification and Characterization of the CaTrxh-Type Family in Pepper

**Figure 1:**
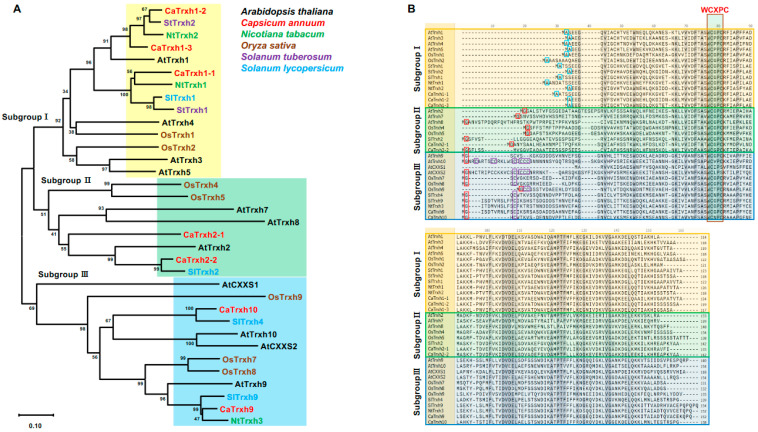
Phylogenetic analysis and multiple sequence alignment of hot pepper CaTrxh-type proteins. (**A**) Phylogenetic analysis of the CaTrxh-type family in five plant species. Phylogenetic analysis was performed in MEGA-X, and trees were generated using the neighbor joining method with a bootstrap of 1000 cycles. Trxh proteins are divided into three subgroups as indicated. (**B**) Protein sequence alignment analysis of hot pepper CaTrxh-type with other Trxh-types. Predicted N-α-acetylation Ala-2 in subgroup I is indicated in blue boxes. In subgroups II and III, Gly-2, which undergoes N-myristoylation, is indicated in red boxes. N-terminal Cys residue in subgroup III, predicted to be palmitoylated, is indicated in purple boxes. The active-site Cys residues and the catalytic motif are indicated by ‘WCXPC’.

While studies on Trx in various crops exist, research on the Trxh family in peppers is still lacking. Therefore, we first identified the Trxh-type family in peppers. Putative hot pepper Trxh-type members were identified using BLAST searches against the genome sequence for hot pepper *C. annuum* cv. CM334 and Zunla-1 (Table 1). We found seven *CaTrxh*-type genes through pepper genome analysis. The Trx domain structure is annotated by SMART (http://smart.embl-heidelberg.de/smart/batch.pl, accessed on 1 January 2023). The molecular weights and theoretical isoelectric points of each CaTrxh-type protein were analyzed using ExPASy (https://www.expasy.org/, accessed on 1 January 2023) (Table 2). From the identification data, we identified seven CaTrxh-type members. To confirm the phylogenetic relationship between Arabidopsis AtTrxh, which is well known for its function, and other important crop plants, a phylogenetic tree was constructed for the Trxh-type protein sequence. As shown in Figure 1A, CaTrxh family proteins were divided into three subgroups according to the classification of AtTrxh. OsTrxh4, OsTrxh5, OsTrxh7, and OsTrxh8 are considered redundant types. These results indicate that the functional and evolutionary properties of CaTrxh present in Solanaceae crops may be similar.

Among the properties of the protein, the N-terminus is highly conserved with glycine in Trxh subgroups II and III. The glycine involved in N-myristoylation was not clearly present in subgroup I (Figure 1B). These results indicate that differences in post-translational modifications between group I and groups II-III result in functional diversity. It has been known that the attachment of myristoyl groups is essential for proteins involved in a variety of biological functions such as subcellular localization and signal transduction [24,25]. Furthermore, subgroup I had putative N-α-acetylation Ala-2 residue. In subgroup III, Trxh-type proteins contained both putative Cys residues for palmitoylation and Gly residues for myristorylation (Figure 1B) [26]. CaTrxh-type proteins also exhibited highly conserved features of N-terminal modifications. This indicated that the N-terminal extension of CaTrxh-type proteins which has been discovered so far may play a more diverse role via post-translational modification. In CaTrxh-type proteins, the active site Cys residue and catalytic motif ‘WCXPC’ were completely conserved (Figure 1B).

### 2.2. Cis-Regulatory Element Analysis of CaTrxh-Type Gene Promoter

**Figure 2:**
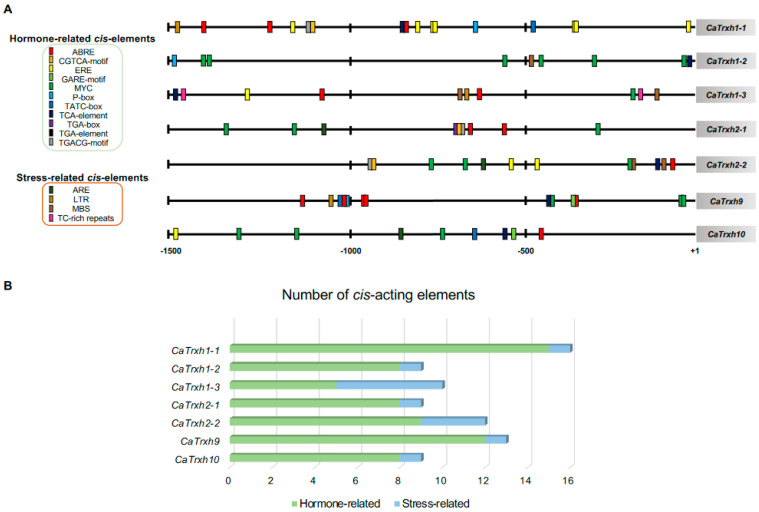
Promoter analysis of *CaTrxh*-type genes. (**A**,**B**) The 1.5 kb promoter of *CaTRX h*-type genes was analyzed via PlantCARE. Important plant hormone-related motifs and stress-related motifs were placed in the promoters using various shapes.

To understand the function of the *CaTrxh* family, we obtained a 1.5 kb promoter region of *CaTrxh*-type genes and analyzed the *cis*-acting regulatory element motifs (Table 3). It was found that motifs mainly responding to plant hormones were distributed in the promoter (Figure 2A,B). Abscisic acid (ABA)-responsive elements such as ABRE and MYC motifs were present in significant numbers of *CaTrxh* promoters. Basically, ABA regulates the regulation of these diverse processes, including physiological responses to cold, drought, and salinity stresses [27,28]. ABRE, an ABA response element belonging to the G-box family, contains an ‘ACGT’ sequence known to be recognized by the plant bZIP transcription factor [29,30]. Interestingly, five ABRE motifs were found in the promoter of *CaTrxh9* (Figure 2A). Several MYC motifs are known to respond to cold stress and involved in regulating *CBF* gene by R2R3-type MYB transcription factors [31,32]. Six MYC motifs were found in the promoter of *CaTrxh1-2* and were abundantly included in the promoters of other *CaTrxh* genes (Figure 2A). Additionally, cis-acting elements related to the cold-induced expression (LTR) motif, MYB-binding motif, and drought-inducible (MBS) motif were each found to be associated with abiotic stress. Thus, we speculated that the LTR and MBS motifs might play a role in regulating the expression of *CaTrxh* genes in response to cold and drought stresses.

### 2.3. Tissue-Specific Gene Expression of CaTrxh Family

**Figure 3:**
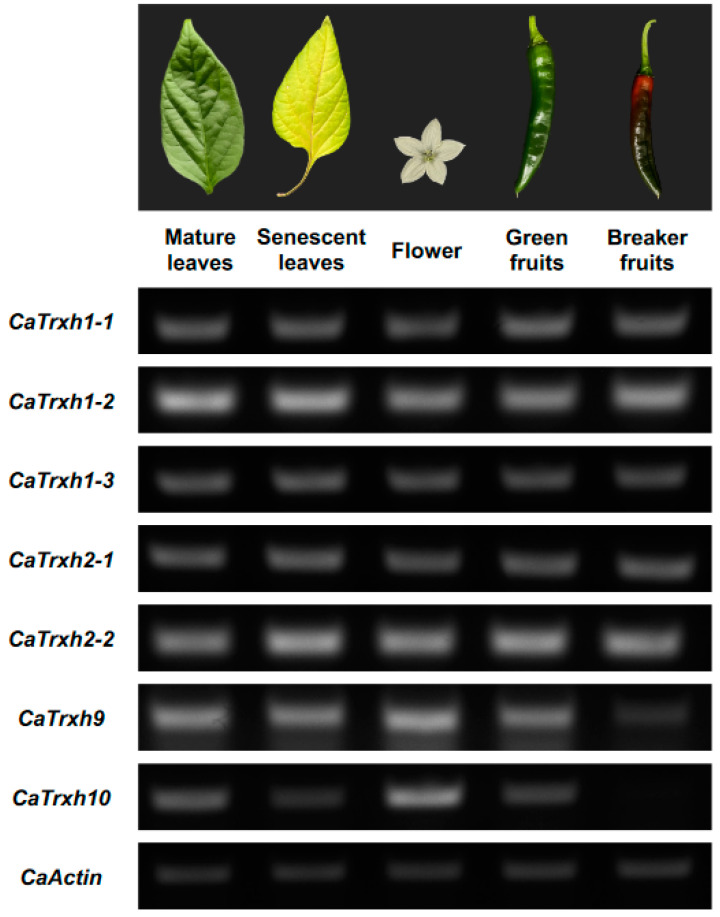
Expression patterns of *CaTrxh* genes in different developmental stages and tissues. Gene expression of the *CaTrxh*-type family was analyzed in mature leaves (ML), senescent leaves (SL), flower (F), green fruit (GF), and breaker fruit (BF) by RT-PCR. The *CaAct* gene was used as a control.

To gain further insight into the development and functional role of *CaTrxh*-type in each tissue, the expression pattern of *CaTrxh*-type was investigated via semi-quantitative RT-PCR. Gene expression of the CaTrxh-type family was confirmed in mature leaves (ML), senescent leaves (SL), flower (F), green fruit (GF), and breaker fruit (BF). All *CaTrxh* types was expressed in ML, SL, F, GF, and BF. *CaTrxh1-1*, *CaTrxh1-2*, *CaTrxh1-3*, *CaTrxh2-1*, and *CaTrxh2-2* showed little change in expression levels; whereas subgroup III *CaTrxh9* and *CaTrxh10* showed reduced gene expression levels in BF (Figure 3). For *CaTrxh10*, gene expression is reduced in SL compared to ML. Thus, some *CaTrxh* genes are expected to affect developmental processes.

### 2.4. Expression Analysis of CaTrxh Genes in Response to Heat and Cold Stresses

**Figure 4:**
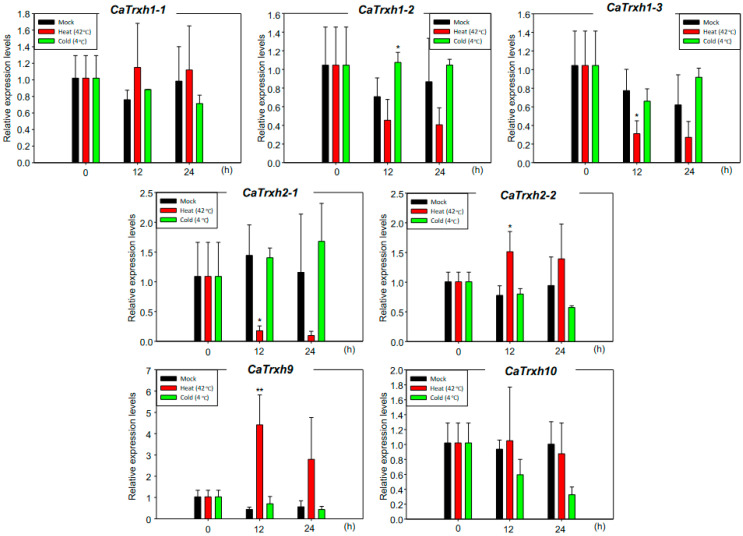
Analysis of gene expression patterns of the *CaTrxh* family under low- and high-temperature stress. Peppers grown for 4 weeks were exposed to stress at 4 °C and 42 °C for 12 h and 24 h, respectively, and gene expression was tested by qRT-PCR. The *CaAct* gene was used as a control. Error bars indicate the standard deviations of three independent qRT-PCR biological replicates. Asterisks indicate significant differences from the control using the unpaired Student’s *t*-test (* *p* < 0.05 and ** *p* < 0.01).

The *Trxh* genes have been reported to be involved in oxidative tolerance in abiotic stress [33,34]. We therefore investigated the gene expression of the *CaTrxh* family in response to cold and heat stresses. *CaTrxh1-3*, *CaTrxh2-1*, *CaTrxh2-2*, and *CaTrxh9* responded to heat stress at 12 h. The expression levels of *CaTrxh1-3* and *CaTrxh2-1* in pepper decreased under heat stress compared to the mock control (Figure 4). The expression levels of *CaTrxh9* were highly increased under heat stress at 12 h (Figure 4). However, transcript levels of *CaTrxh1-1*, *CaTrxh1-2*, and *CaTrxh10* were not significantly changed under both stresses.

### 2.5. Subcellular Localization of the CaTrxh Family

**Figure 5:**
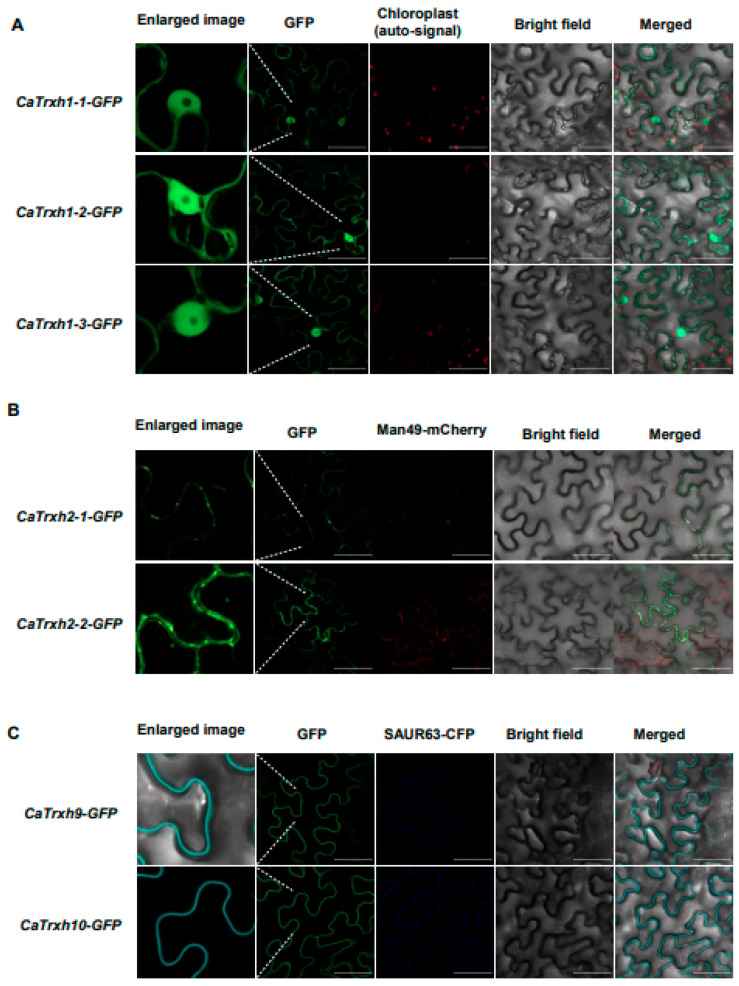
Analysis of various subcellular localizations of the CaTrxh-type family. Each *CaTrxh* gene was cloned into a *35S::GFP* vector (C-terminal GFP fusion), and *Agrobacterium*-mediated transient assay was performed in *N. benthamiana*. After infiltration, the GFP signal was checked using a confocal microscope. (**A**) Subcellular localization of subgroup I members: CaTrxh1-1-GFP, CaTrxh1-2-GFP, and CaTrxh1-3-GFP. The GFP signal of a member of group I was merged with the chloroplast auto-signal (Red). (**B**) Subcellular localization of subgroup II members: CaTrxh2-1-GFP and CaTrxh2-2-GFP. The GFP signal of a member of group II was merged with Man49-mCherry, a Golgi marker. (**C**) Subcellular localization of subgroup III members: CaTrxh9-GFP and CaTrxh10-GFP. The GFP signal of group III member was merged with SAUR63-CFP, a plasma membrane marker. Scale bar: 50 µm.

Trxh proteins present in plants have evolved various isoforms involved in the redox regulation of a wide range of metabolic processes in various intracellular compartments [35]. The subcellular localization of Trxh family proteins with different targets or substrates makes significant differences [36,37]. The subcellular localization of Trxh-type in crops is not yet well understood. Therefore, we decided to fuse *CaTrxh* genes with GFP to determine their subcellular localization. As shown in Figure 5A, the GFP signals of *35S::CaTrxh1-1-GFP*, *35S::CaTrxh1-2-GFP*, and *35S::CaTrxh1-3-GFP*, classified into subgroup I, were equally observed in the cytoplasm and nucleus. Thus, subgroup I of CaTrxh is predicted to have a similar function not only in the cytoplasm but also in the nucleus.

In subgroup II, OsTrxh4 has been identified as an interactor of rice phosphate overaccumulator 2 (OsPHO2), which is an E2 ubiquitin conjugase, to regulate phosphate homeostasis. Interestingly, subcellular localizations of OsTrxh4 were detected mainly in the cytoplasm and ER/Golgi membrane [38]. To further confirm their subcellular localization, *35S::CaTrxh2-1-GFP* and *35S::CaTrxh2-2-GFP* were expressed together with Golgi-resident fusion protein (Man49-mCherry), respectively. GFP signals of *35S::CaTrxh2-1-GFP* and *35S::CaTrxh2-2-GFP* were perfectly merged with Golgi marker (Figure 5B and Figure S2). Although the effective reducing agents of chloroplasts are mainly known as Trx y, Trx x, Trx m, and Trx f [39], the CaTrxh-type family was not located in chloroplasts. For subgroup II, GFP signals were occasionally detected in the cytoplasm and the nucleus (Figure S1A). In the case of subgroup III, GFP was observed not only in the cell membrane but also weakly in the nucleus (Figure S1B). The subcellular localization of CaTrxh is believed to be highly variable. In subgroup III, GFP signals of *35S::CaTrxh9-GFP* and *35S::CaTrxh10-GFP* were merged with plasma membrane (PM)-localized SAUR63-CFP marker (Figure 5C). Likewise, the subcellular localization of AtTrxh9 was at the plasma membrane [40]. SlTrxh9 and NtTrxh9 proteins formed a small clade in subgroup III (Figure 1A). This clade was expected to have specialized functions in the cell membrane through N-myristoylation.

### 2.6. CaTrxh Protein Changes in Subcellular Localization under Heat Stress

It has been reported that under cold stress conditions, demyristoylation of cytoplasmic AtTrxh2 allows for intracellular translocation into the nucleus and the subsequent dissociation from CBF oligomers, a transcriptional regulator [41]. As shown in Figure 1, Trxh-type subgroups II and III had characteristics of N-myristoylation, which showed a conserved Gly residue in position 2. These biochemical properties of N-myristoylation are known to alter their intracellular localization [26]. Furthermore, subgroup III contained conserved N-terminal Cys residue to be palmitoylated (Figure 1B). This Cys residue is also known to be an important modification site for plasma membrane localization [26].

**Figure 6:**
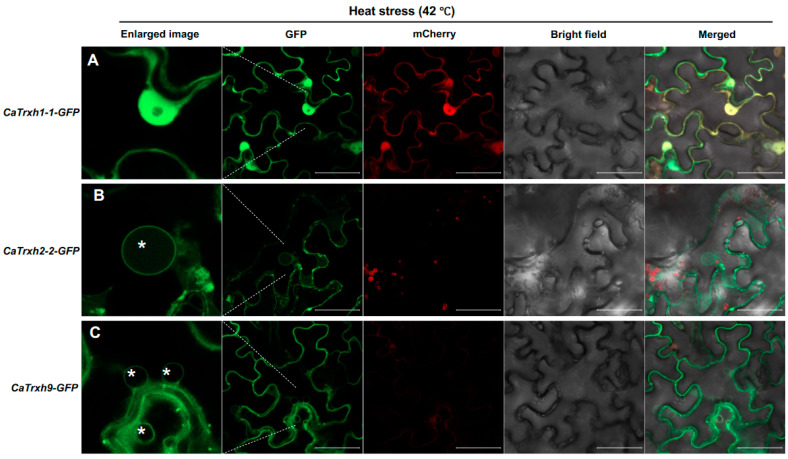
CaTrxh-type proteins undergo changes in their subcellular localization in response to heat stress. (**A**–**C**) *35S::CaTrxh1-1-GFP*, *35S::CaTrxh2-2-GFP*, and *35S::CaTrxh9-GFP* were transiently expressed in *N. benthamiana* leaves for 3 days. The plants were then incubated at 42 °C for 1 h to examine their subcellular localization. *35S::mCherry* vector used as a negative control upon the heat stress. The asterisk indicates the tonoplast. Scale bar: 50 µm.

Based on the results that the *CaTrxh2-2* and *CaTrxh9* genes’ response to heat stress (Figure 4), we hypothesized that heat stress may cause changes in the subcellular localization of CaTrxh protein. CaTrxh1-1-GFP showed no change in its subcellular location under high-temperature stress (Figure 6A). For CaTrxh2-2-GFP and CaTrxh9-GFP, their intracellular localization to the tonoplast was changed under high-temperature conditions (Figure 6B,C). CaTrxh9-GFP was mainly observed in the plasma membrane, and under high-temperature stress, its intracellular location was observed to change to various nucleus, tonoplast, and spot-shaped punctate organelles (Figure 6C). This spot-like intracellular location of CaTrxh9-GFP or CaTrxh10-GFP was not merged with Man46-mCherry and was not in the Golgi (Figure S3). These results predict that CaTrxh-type proteins regulate the redox of target proteins at various intracellular locations to overcome high-temperature stress.

### 2.7. CaTrxh Proteins Were Able to Make Complexes via Homomeric Association

**Figure 7:**
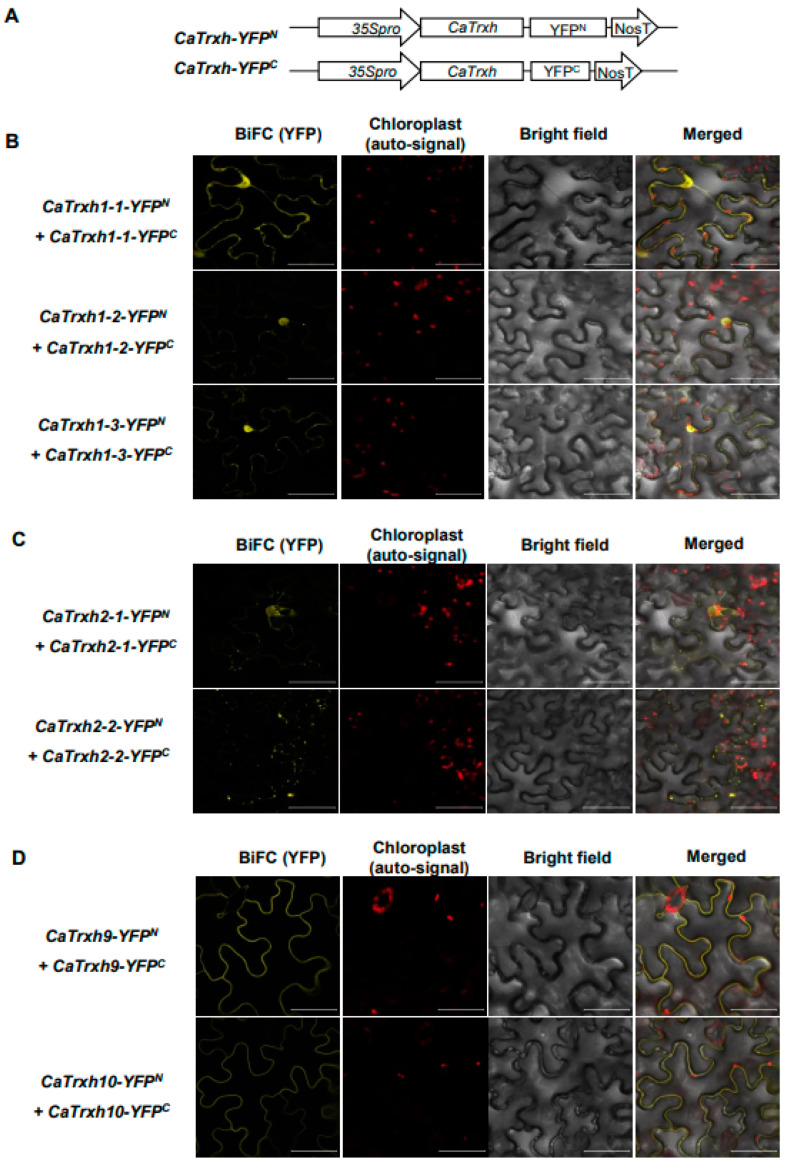
All CaTrxh-type proteins form homodimers in BiFC assay. (**A**) Construction of split-YFP. (**B**) In group I, members were observed to have homodimer interactions in the BiFC assy. Reconstituted YFP signals were observed in the combinations of CaTrxh1-1-*YFP^N^*/CaTrxh1-1-*YFP^C^* or CaTrxh1-2-*YFP^N^*/CaTrxh1-2-*YFP^C^* or CaTrxh1-3-*YFP^N^*/CaTrxh1-3-*YFP^C^*. BiFC analysis was performed on an *Agrobacterium*-mediated transient gene expression system. (**C**) In group II, *CaTrxh2-1-YFP^N^* and *CaTrxh2-1-YFP^C^* or *CaTrxh2-2-YFP^N^* and *CaTrxh2-2-YFP^C^* constructs were coexpressed in *N. benthamiana*. These CaTrxh group II showed YFP fluorescence. (**D**) In group III, *CaTrxh9-YFP^N^* and *CaTrxh9-YFP^C^* or *CaTrxh10-YFP^N^* and *CaTrxh10-YFP^C^* constructs were coexpressed in *N. benthamiana*. Scale bar: 50 µm.

Trx protein is an important intracellular protein that regulates protein activity and is known to interact with various targets [13,42]. However, since research on the interaction between thioredoxin family proteins has not yet been conducted, we used bimolecular fluorescence complementation (BiFC) analysis to determine the interaction between the CaTrxh-type protein family (Figure 7A). In CaTrxh subgroup I, combinations of *CaTrxh1-1-YFP^N^* and *CaTrxh1-1-YFP^C^* or *CaTrxh-1-2-YFP^N^* and *CaTrxh1-2-YFP^C^* or *CaTrxh1-3-YFP^N^* and *CaTrxh1-3-YFP^C^* were transiently expressed in tobacco plants. The reconstituted YFP signals were observed in the nucleus and cytoplasm (Figure 7B). The results showed that all CaTrxh subgroups I formed homodimers. Similarly, homodimerization of subgroup II members CaTrxh2-1 and CaTrxh2-2 was tested. The YFP signals detected that CaTrxh2-1-YFP^N^ and CaTrxh2-1-YFP^C^ or CaTrxh2-2-YFP^N^ and CaTrxh2-2-YFP^C^ each formed a homodimeric complex in the Golgi region (Figure 7C). CaTrxh9 and CaTrxh10, corresponding to group III, each formed a homodimeric complex in the plasma membrane (Figure 7D). Thus, CaTrxh family proteins within each group will function through homodimerization.

### 2.8. CaTrxh Proteins Exhibited Heteromeric Protein Complexes

**Figure 8:**
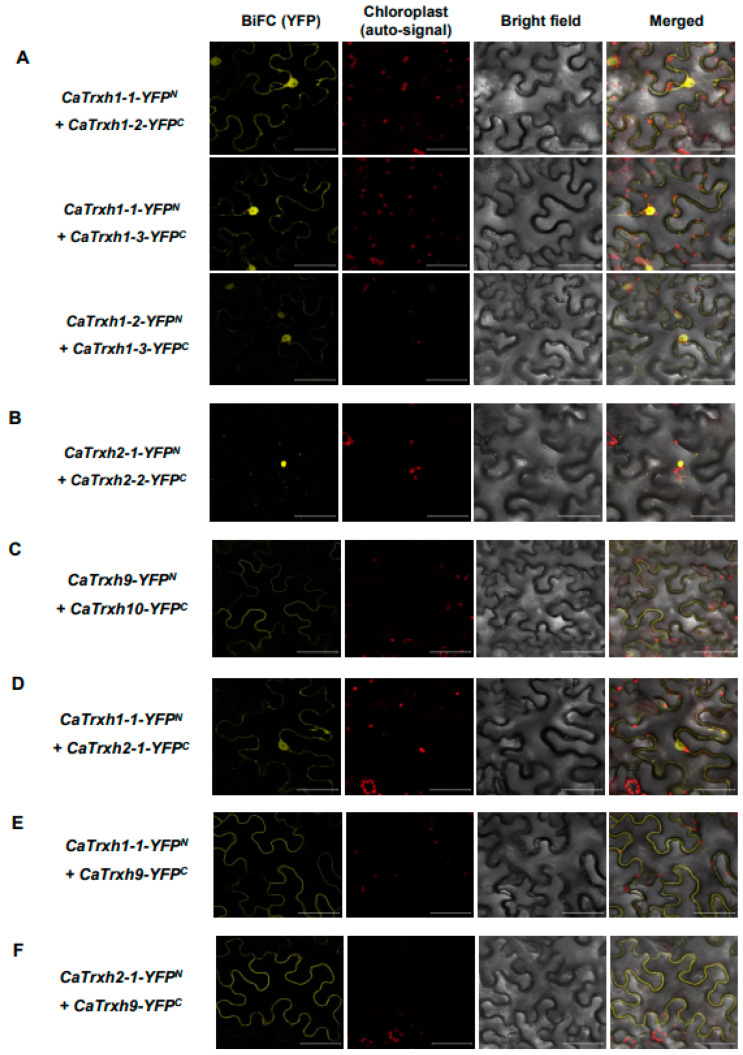
All CaTrxh-type proteins make heterodimers in BiFC assay. (**A**) In group I, CaTrxh protein make a heteromeric complex in BiFC. The *CaTrxh1-1*-*YFP^N^*/*CaTrxh1-2*-*YFP^C^* or *CaTrxh1-1*-*YFP^N^*/*CaTrxh1-3*-*YFP^C^* or *CaTrxh1-2*-*YFP^N^*/*CaTrxh1-3*-*YFP^C^* combinations were transiently expressed in tobacco leaves. (**B**) In group II, *CaTrxh2-1-YFP^N^* and *CaTrxh2-2-YFP^C^* constructs were coexpressed in *N. benthamiana*. (**C**) In group III, *CaTrxh9-YFP^N^* and *CaTrxh10-YFP^C^* constructs were coexpressed in *N. benthamiana*. (**D**) In group I and group II combinations, *CaTrxh1-1-YFP^N^* and *CaTrxh2-1-YFP^C^* constructs showed reconstructed YFP signal when coexpressed. (**E**). In group I and group III combinations, *CaTrxh1-1-YFP^N^* and *CaTrxh9-YFP^C^* constructs were coexpressed and YFP signal was observed. (**F**). In group II and group III combinations, *CaTrxh2-1-YFP^N^* and *CaTrxh9-YFP^C^* constructs were coexpressed. Scale bar: 50 µm.

We previously showed through BiFC that CaTrxh proteins form homodimerization with each other. Then, since CaTrxh exists in the same intracellular location within each group, we hypothesized that it may function through a heterodimer. The combination of CaTrxh1-1-YFP^N^ and CaTrxh1-2-YFP^C^ or CaTrxh1-1-YFP^N^ and CaTrxh1-3-YFP^C^ or CaTrxh1-2-YFP^N^ and CaTrxh1-3-YFP^C^, corresponding to group I, was transiently expressed in tobacco plants. All BiFC combinations resulted in reconstructed YFP signals (Figure 8A). As shown in Figure 8B.C, heterodimeric complexes were also formed in group II and group III. These results are expected to indicate that CaTrxh-type proteins with similar subcellular localization within the group perform functional complementation through homo-/heterocomplexes.

We then hypothesized whether CaTrxh, which has different subcellular localizations, forms heterodimeric complexes through protein interactions. To test this, we selected CaTrxh from each group and performed BiFC. Interestingly, strong YFP signals were observed partially in the Golgi region and nucleus in the group 1 *CaTrxh1-1-YFP^N^* and group 2 *CaTrxh2-1-YFP^C^* (Figure 8D). Through heterodimerization of different groups of Catrxh proteins, the intracellular localization of heteromeric CaTrxh proteins is altered. Similarly, we confirmed that group III CaTrxh9, which mainly exists in the plasma membrane, moves group I CaTrxh1-1 or group II Catrxh2-1 to the cell membrane (Figure 8E,F). These results show that the intracellular location of CaTrxh can be regulated in various ways by forming homodimeric-/heterodimeric complexes and are expected to affect target protein regulation.

### 2.9. Thioredoxin Protein Activity Is Not Related with CaTrxh Protein Complex and Relocalization

**Figure 9:**
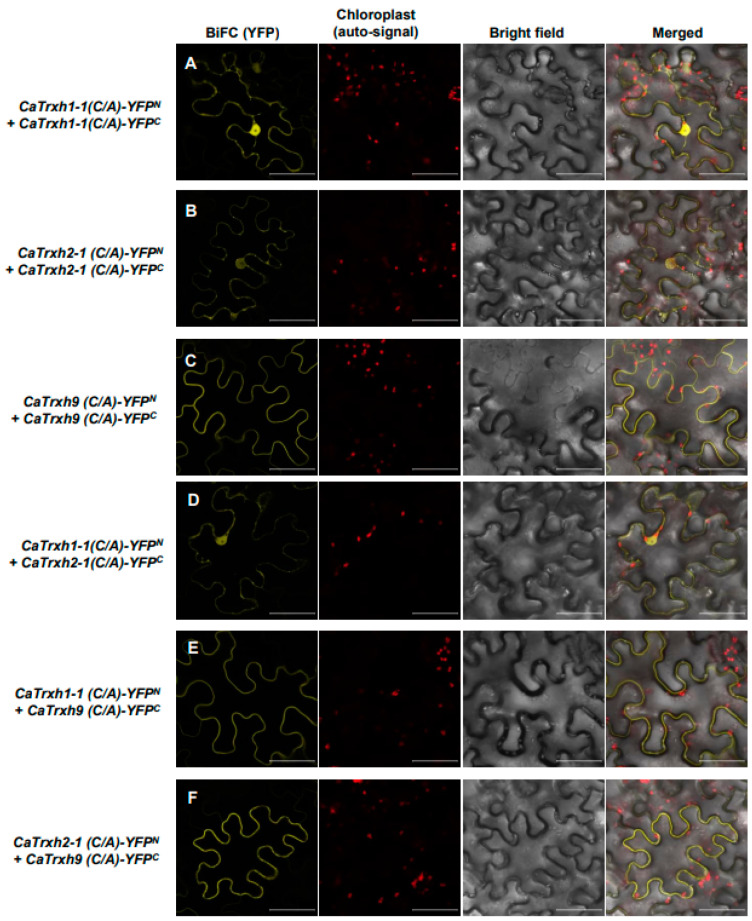
Cysteine active motif mutation does not affect homodimer and heterodimer interactions in the CaTrxh family. (**A**–**C**) Mutations in the cysteine active motif of CaTrxh family proteins do not affect homodimerization and subcellular localization. *CaTrxh1-1(C/A)-YFP^N^* and *CaTrxh1-1(C/A)-YFP^C^* or *CaTrxh2-1(C/A)-YFP^N^* and *CaTrxh2-1(C/A)-YFP^C^* or *CaTrxh9(C/A)-YFP^N^* and *CaTrxh9(C/A)-YFP^C^*, in which two cysteine residues of the CXXPC motif were mutated, were transiently expressed in tobacco, and were observed via confocal microscopy. (**D**–**F**) Intergroup heterodimer formation and intracellular localization of CaTrxh family proteins with cysteine active motif mutations were not affected. *CaTrxh1-1(C/A)-YFP^N^* and *CaTrxh1-1(C/A)-YFP^C^* or *CaTrxh2-1(C/A)-YFP^N^* and *CaTrxh2-1(C/A)-YFP^C^* or *CaTrxh9(C/A)-YFP^N^* and *CaTrxh9(C/A)-YFP^C^* were coexpressed in tobacco plants, and the reconstituted YFP signals were observed using confocal microscopy. Scale bar: 50 µm.

Since it is already well known that the N-myristoylation site is an important factor in determining the intracellular localization of Trxh, we tested whether mutations in the redox-active disulfide motif, which is an important part of CaTrxh enzymatic activity, affect its subcellular localization and protein interactions. First, the Cys residue of the catalytic motif ‘WCXPC’ mutated to ‘WAXPA’. *CaTrxh1-1(C/A)-GFP*, *CaTrxh2-1(C/A)-GFP*, and *CaTrxh9(C/A)-GFP* constructs were transiently expressed in *N. benthamiana*. The results showed that mutations in the catalytic motif did not affect subcellular localization (Figure S4). We tested whether mutations in the catalytic motif affected the formation of homodimers and heterodimers. As shown in Figure 9A–C, the homodimer of CaTrxh was observed normally without any intracellular changes. In the case of the heterodimer of CaTrxh, *CaTrxh2-1(C/A)-YFP^N^* corresponding to group II interacted with *CaTrxh1-1(C/A)-YFP^C^* of group I, resulting in a strong YFP signal in the Golgi and nucleus (Figure 9D). Group I *CaTrxh1-1(C/A)-YFP^N^* or group II *CaTrxh2-1(C/A)-YFP^N^* formed a strong YFP signal at the plasma membrane with *CaTrxh9(C/A)-YFP^C^* (Figure 9E,F). As a result, the cysteine catalytic motif is an important region in redox regulation but is not related to the homo-/heterocomplex or intracellular localization of CaTrxh.

**Figure 10:**
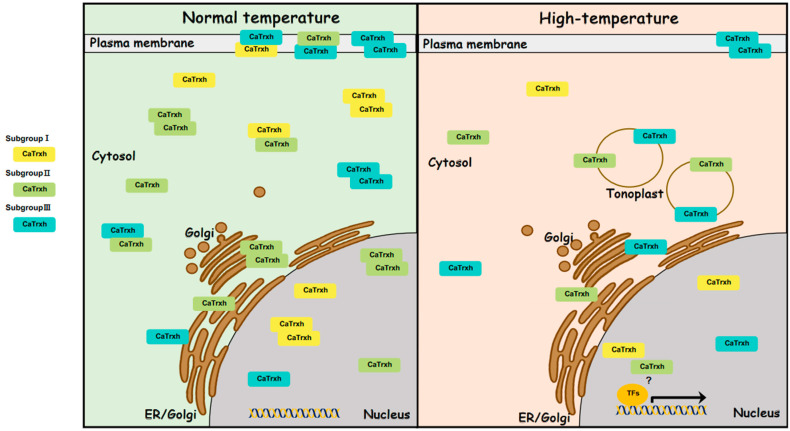
A model for subcellular localization and protein–protein interactions of the CaTrxh family under heat stress. The CaTrxh-type family is largely divided into three groups, and their intracellular locations are quite specific subcellular localizations. Subgroup I is located in the nucleus and cytoplasm. Subgroup II is predominantly located in the Golgi apparatus and cytosolic regions. Subgroup III is mainly located in the plasma membrane. Interestingly, all CaTrxh family proteins can form homodimers and heterodimers. In the BiFC results of subgroup III and subgroup I or subgroup II present in the plasma membrane, these CaTrxh subgroup III proteins can move subgroup I or subgroup II proteins to the cell membrane. Additionally, when exposed to heat stress, subgroup I showed no change in intracellular location, whereas subgroup II and subgroup III showed a shift toward the tonoplast. This intracellular localization is expected to play a role in regulating the redox form of various target proteins under high-temperature stress or protecting cells through chaperone function. ‘?’ indicates the possibility that CaTrxh directly regulates the activity of a transcription factor (TF).

We found that some CaTrxh proteins have altered intracellular localization upon high-temperature stress. And the function of CaTrxh proteins does not seem to depend on the catalytic motif. The diverse intracellular locations and relocalizations of CaTrxh through protein interactions result in the production of a variety of intracellular target proteins. Based on our results, the regulatory actions of CaTrxh are briefly presented in Figure 10.

## 3. Discussion

Trx proteins present in plants are largely divided into six major clusters based on Arabidopsis. Among them, the *Trxh*-type has a variety of isotypes compared to other *Trx* family genes. In a representative functional study, biological and abiotic stresses on Arabidopsis AtTrxh2 and AtTrxh5 were known. These Trxh-type proteins have disulfide reductase activity as well as chaperon activity or denitrosation activity [18,19]. These results lead to interest in the functions of the Trxh-type present in various crops [43]. *CaTrxh*-type is highly conserved in Solanaceae crops such as tomato, potato, and tobacco. Some of the *CaTrxh* genes showed a tendency to decrease or increase gene expression under heat stress (Figure 4). Thus, *CaTrxh*-type family could play an essential role in abiotic stresses. Additionally, CaTrxh family proteins were localized in the cytoplasm and other subcellular organelles. There were CaTrxh-type proteins present at specific subcellular locations in the cell. This study serves as the basis for functional studies of the CaTrxh-type family, which is essential for future developments in crops with improved stress responses. CaTrxh-type proteins are identified as potential antioxidant proteins or chaperon-like proteins.

Although Trxh-type mainly presented in the cytoplasm, the intracellular localization of Trxh-type protein is not considered critical. Recently, it has been reported that the perennial plant (*Saussurea involucrata*) SikTrxh, belonging to h-type subgroup I, is completely present in chloroplasts [44]. AtTrxh2 and AtTrxh9 are also present in the mitochondria and plasma membrane, respectively [40]. Similarly, we also found that CaTrxh9-GFP is localized to the plasma membrane and CaTrxh2-2-GFP in located in the Golgi and cytosolic regions (Figure 5). The intracellular localization of Trxh-types can be predicted but cannot be specified, and their functions will also be more diversified. On the other hand, it can be predicted that Trx proteins of similar groups in the phylogenetic tree will have almost-similar subcellular localizations or functions. For example, loss-of-function mutant phenotypes of *AtTrxh9* showed strong dwarfism and developmental defects [40]. If the *CaTrxh9* gene is suppressed by virus-induced gene silencing (VIGS) in hot pepper, it will be developmentally deficient, similar to the *AtTrxh9* mutant. Furthermore, membrane-localized Trxh-type proteins could associate with a target protein different from that of cytosolic Trxh proteins.

There are some reports that Trxh is a useful genetic resource for overcoming various oxidative stresses. In other words, the enzymatic properties of redox regulation, which are specialized for photosynthesis, seem to form various isotypes in plants as a way to overcome additional environmental stress. Recently, AtTrxh2 have been shown to regulate CBF transcription factor activity upon cold stress. It was reported that AtTrxh2 protein and mRNA expression levels were not different under cold stress or warm conditions [41]. Unlike Arabidopsis, CaTrxh-type genes present in pepper did not respond significantly to cold stress. However, we found that *CaTrxh2-1*, *CaTrxh2-2*, and *CaTrxh9* genes response to high temperature. Thus, the *CaTrxh*-type gene would be functionally related to climate change phenomena such as temperature stress. AtTrxh3 has chaperone activity, and transgenic Arabidopsis plants overexpressing it showed improved heat stress tolerance even without disulfide reduction activity [45]. Our results showed that mutations in the CaTrxh cysteine active motif did not affect the subcellular localization or protein interactions. The CaTrxh-type family can act as both chaperones and disulfide reductases and play an important role in preserving the functional integrity of key target proteins; in particular, its function will be even more important under oxidative stress conditions.

Many members of the *Trxh*-type gene family and their diverse subcellular locations may be closely related to their functions. The most effective approach to engineering crops to overcome climate change is to adopt methods that enhance tolerance to oxidative stress [43]. In this study, we identified seven *CaTrxh*-types genes and confirmed that *CaTrxh*-type genes respond to abiotic stress. We also found that CaTrxh proteins were present at specific subcellular locations in plant cells and that their locations changed in response to heat stress (Figure 4). It is necessary to make transgenic plants using the *CaTrxh* gene and to test what kind of phenotype they have in response to high-temperature stress. *CaTrxh*-type genes may have a useful high-temperature tolerance phenotype.

Recently, the THIOREDOXIN INTERACTING RECEPTOR KINASE (TIRK) transmembrane receptor was discovered as a new target for AtTrxh5. AtTrxh5 modulates the S-nitrosylation of TIRK and participates in redox-dependent regulation of this receptor [46]. In particular, it is well known that NPR1 undergoes structural changes through protein interaction with AtTrxh5 and migrates to the nucleus to activate defense [17,47]. We therefore wondered if CaTrxh-family protein could interact with CaNPR1 present in pepper, and whether this is a specific and evolutionally conserved regulation mechanism. Consequently, the diversified *CaTrxh*-type family is thought to have various strategies for coping with oxidative stress in crops.

## 4. Materials and Methods

### 4.1. Plant Materials

Seeds of hot pepper (*Capsicum annuum* L. cv. Bugang) and *Nicotiana benthamiana* were sown in commercial bed soil. After the germination of pepper or tobacco seeds on a plate with 100% moisture, the plants were individually transplanted into 500 mL pots. The plants were cultivated under the following conditions: a growth room/growth chamber with a 16 h LED light (color temperature ranging from 5000 K to 6500 K) and an 8 h dark photoperiod at 22 ± 2 °C with 60–70% relative humidity. Four-week-old seedlings were utilized for the subsequent treatments.

### 4.2. Sample RNA Extraction, Semi-Quantitative RT-PCR, and qRT-PCR

Transcriptional responses of *CaTrxh*-types upon heat (42 °C) and cold (4 °C) treatments. For heat and cool treatments, hot pepper plants were grown for 4 weeks, provided with enough water, and then treated with mock (22 °C), heat (42 °C), and cold (4 °C) for 0, 12, and 24 h. Three biological replicate samples were mRNA extracted via isolRNA kit (TaKaRa, Kusatsu, Japan) in accordance with the commercial protocol. Briefly, the sample tissue was rapidly frozen using liquid nitrogen (LN2) and subsequently finely ground with a mortar and pestle. Following this, 1000 μL of Trizol RNAiso solution was added, thoroughly mixed, and left at room temperature for 20 min. The mixture was then centrifuged at 13,000 rpm and 4 °C for 5 min. The upper layer was carefully transferred to a new tube, and 500 μL of isopropanol was added. After incubating at room temperature for 10 min, the solution was centrifuged at 13,000 rpm for an additional 10 min at 4 °C to obtain the RNA pellet. For cDNA synthesis, total RNA 5 µg was reacted with MMLV cDNA synthesis kit (ELPIS BIO). For qPCR experiments, SYBR Green kit (Biorad and KAPAbio) was used. Data normalization was performed with a reference gene, *CaActin*. All primers used for semi-quantitative RT-PCR and qPCR are presented in Supplementary Table S1.

### 4.3. Identification, Protein Sequence Alignment, and Phylogenetic Tree of CaTrxh-Type Subfamily

*CaTrxh*-type genes were manually searched and selected via the Solanaceae Genomics Network (https://solgenomics.net/, accessed on 12 December 2022) and Pepper genome (http://peppergenome.snu.ac.kr/blast.php, accessed on 20 December 2022). Molecular weight and isoelectric point of pepper CaTrxh-type proteins were calculated by a protein isoelectric point calculator. CaTrxh-type protein sequences were aligned with identified Arabidopsis, tobacco, and tomato Trxh protein sequences via the neighbor joining method. Phylogenetic analysis was performed in MEGA-X [48].

### 4.4. Plasmid Construct of CaTrx-h-Type Genes and Site-Direct Mutagenesis

Vector constructs of *35S::CaTrxh-GFP, 35S::SAUR63-CFP, 35S::CaTrxh-YFP^N^,* and *35S::CaTrxh-YFP^C^* were generated using pGPTV-II-GFP, pGPTV-II-CFP, pSPYNE, and pSPYCE vectors with appropriate restriction enzymes [49]. The inactive mutant ‘WAGPA’ was generated by replacing cysteine (C) in ‘WCGPC’, the conserved active site of thioredoxin, with alanine (A). Experiments were performed using EZchange™ Site-directed Mutagenesis Kit (Enzynomics, Daejeon, Republic of Korea). Primers were created using NEBaseChanger (https://nebasechanger.neb.com/, accessed on 15 September 2023). Cloning primers are listed in Supplementary Table S1.

### 4.5. BiFC Analysis and Subcellular Localization Analysis

For the BiFC assay, the *CaTrxh* gene was cloned into pSPYNE and pSPYCE vectors, each containing a split YFP at the C-terminus, and subsequently transformed into *Agrobacterium tumefaciens* GV3101. *Agro*-cells containing the *35S::CaTrxh-YFP^N^* and *35S::CaTrxh-YFP^C^* constructs were co-delivered to tobacco leaves. The reconstructed YFP signal was observed via confocal microscopy. Additionally, to determine the subcellular localization of the CaTrxh protein, all genes of the *CaTrxh*-type family were fused with GFP at the N-terminus.

For transient expression analysis, *A. tumefaciens* GV3101 was used in infiltration. Briefly, *Agrobacterium* cells (OD_600_ = 0.5–1.0) containing the appropriate vector construct were syringe-infiltrated with infiltration buffer (10 mM MgCl_2_, 10 mM MES, pH 5.6) in 3–4 week-old *Nicotiana benthamiana* leaves [50]. After 3-day post infiltration, YFP (excitation 513 nm–emission 527 nm), GFP (excitation 489 nm–emission 509 nm), chloroplast autofluorescence (excitation 640 nm–emission 680 nm), mCherry (excitation 587 nm–emission 610 nm), and CFP (excitation 300 nm–emission 600 nm) signals were detected via a confocal microscope Carl Zeiss LSM900.

### 4.6. Promoter Analysis of CaTrxh-Type Genes

The 1.5 kb *CaTrxh*-type gene sequences upstream to the start codon were retrieved from Ensembl Plants. The promoter DNA sequence of *CaTrxh*-type genes was analyzed via PlantCARE database (http://bioinformatics.psb.ugent.be/webtools/plantcare/html/, 15 February 2023) [51]. Identified *cis*-element motifs from the promoter sequence of *CaTrxh*-type genes were investigated and summarized in Table 3.

## Figures and Tables

**Table 1:** GeneBank accession numbers of Arabidopsis thaliana, Oryza sativa, Solanum tuberosum, Solanum lycopersicum, Nicotiana tabacum, and Capsicum annuum Trxh-type family.

	Gene	Accession Number
*Arabidopsis thaliana*	*AtTrxh1*	NP_190672.1
	*AtTrxh2*	NP_198811.1
	*AtTrxh3*	NP_199112.1
	*AtTrxh4*	NP_173403.1
	*AtTrxh5*	NP_175128.1
	*AtTrxh7*	NP_176182.1
	*AtTrxh8*	NP_177146.1
	*AtTrxh9*	NP_001078124.1
	*AtTrxh10*	NP_001325846.1
	*AtCXXS1*	NP_172620.1
	*AtCXXS2*	NP_001318396.1
*Oryza sativa*	*OsTrxh1*	XP_015647572.1
	*OsTrxh2*	XP_015640796.1
	*OsTrxh4*	XP_015631704.1
	*OsTrxh5*	XP_015646978.1
	*OsTrxh7*	XP_015625017.1
	*OsTrxh8*	XP_015640764.1
	*OsTrxh9*	XP_015639270.1
*Solanum tuberosum*	*StTrxh1*	XP_006341987.1
	*StTrxh2*	NP_001275313.1
*Solanum lycopersicum*	*SlTrxh1*	XP_004238306.1
	*SlTrxh2*	XP_010316263.1
	*SlTrxh4*	XP_004235220.1
	*SlTrxh9*	XP_004246283.1
*Nicotiana tabacum*	*NtTrxh1*	CAA41415.1
	*NtTrxh2*	Q07090.1
	*NtTrxh3*	XP_016458904.1
*Capsicum annuum*	*CaTrxh1-1*	XP_016568496.1
	*CaTrxh1-2*	XP_016560703.1
	*CaTrxh1-3*	XP_016552903.1
	*CaTrxh2-1*	XP_016548658.2
	*CaTrxh2-2*	XP_016561441.1
	*CaTrxh9*	XP_016541800.1
	*CaTrxh10*	XP_016564926.1

**Table 2:** Prediction of physicochemical properties of *CaTrxh* family.

Gene Name	Accession Number	Chromosome	Length (aa)	Molecular Weight	Theoretical pI	Instability Index	Aliphatic Index	GRAVY	Stability
*CaTrxh1-1*	XM_016713010.1	4	124	13,717.77	5.01	30.8	85.65	0.008	YES
*CaTrxh1-2*	XM_016705217.1	2	119	13,186.19	5.3	25.34	86.81	−0.047	YES
*CaTrxh1-3*	XM_016697417.1	4	117	12,849.94	5.34	24.2	94.19	0.067	YES
*CaTrxh2-1*	XM_016693172.1	11	135	15,286.76	6.96	20.14	88.81	−0.136	YES
*CaTrxh2-2*	XM_016705955.1	2	142	15,655.87	5.23	44.58	81.06	−0.185	NO
*CaTrxh9*	XM_016686314.1	9	152	16,830.12	4.82	30.95	77.7	−0.189	YES
*CaTrxh10*	XM_016709440.1	3	138	15,415.41	5.06	34.65	69.93	−0.463	YES

**Table 3:** Types and numbers of known *cis*-elements in the promoter regions of *CaTrxh*-type genes.

*Cis*-Element	Function	*CaTrxh1-1*	*CaTrxh1-2*	*CaTrxh1-3*	*CaTrxh2-1*	*CaTrxh2-2*	*CaTrxh9*	*CaTrxh10*
**Hormone-related**								
ABRE	*cis*-element involved in the abscisic acid responsiveness	3	-	2	2	1	5	1
CGTCA-motif	*cis*-element involved in the MeJA-responsiveness	1	-	-	1	1	-	-
ERE	ethylene-responsive element	7	-	1	-	2	-	1
GARE-motif	gibberellin-responsive element	-	-	-	-	-	1	1
MYC	*cis*-acting element involved in the abscisic acid responsiveness	-	6	1	3	3	3	3
P-box	gibberellin-responsive element	1	1	-	-	-	1	-
TATC-box	*cis*-element involved in gibberellin-responsiveness	1	-	-	-	-	1	1
TCA-element	*cis*-element involved in salicylic acid responsiveness	1	1	1	-	1	1	1
TGA-box	part of an auxin-responsive element	-	-	-	1	-	-	-
TGA-element	auxin-responsive element	-	-	-	-	-	-	-
TGACG-motif	*cis*-acting regulatory element involved in the MeJA-responsiveness	1	-	-	1	1	-	-
***Stress-related***								
ARE	*cis*-element essential for the anaerobic induction	-	-	-	1	1	-	1
LTR	*cis*-element involved in low-temperature responsiveness	1	-	1	-	-	1	-
MBS	MYB binding site involved in drought-inducibility	-	1	2	-	2	-	-
TC-rich repeats	*cis*-element involved in defense and stress responsiveness	-	-	2	-	-	-	-

## Data Availability

The data presented in this study are available on request from the authors. Informed consent was obtained from all subjects involved in the study.

## Supplementary Materials

The following supporting information can be downloaded at https://www.mdpi.com/article/10.3390/ijms25031729/s1.

## References

[1] Gao L., Kantar M.B., Moxley D., Ortiz-Barrientos D., Rieseberg L.H. (2023). Crop adaptation to climate change: An evolutionary perspective. Mol. Plant.

[2] Wani K.I., Naeem M., Castroverde C.D.M., Kalaji H.M., Albaqami M., Aftab T. (2021). Molecular Mechanisms of Nitric Oxide (NO) Signaling and Reactive Oxygen Species (ROS) Homeostasis during Abiotic Stresses in Plants. Int. J. Mol. Sci..

[3] Devireddy A.R., Rivero R.M., Zandalinas S.I. (2023). Editorial: Rising stars in plant ROS/redox biology under abiotic stress conditions. Front. Plant Sci..

[4] Meyer Y., Buchanan B.B., Vignols F., Reichheld J.P. (2009). Thioredoxins and glutaredoxins: Unifying elements in redox biology. Annu. Rev. Genet..

[5] Sevilla F., Marti M.C., De Brasi-Velasco S., Jimenez A. (2023). Redox regulation, thioredoxins, and glutaredoxins in retrograde signalling and gene transcription. J. Exp. Bot..

[6] Geigenberger P., Thormahlen I., Daloso D.M., Fernie A.R. (2017). The Unprecedented Versatility of the Plant Thioredoxin System. Trends Plant Sci..

[7] Ji M.G., Park H.J., Cha J.-Y., Kim J.A., Shin G.-I., Jeong S.Y., Lee E.S., Yun D.-J., Lee S.Y., Kim W.-Y. (2020). Expression of Arabidopsis thaliana Thioredoxin-h2 in Brassica napus enhances antioxidant defenses and improves salt tolerance. Plant Physiol. Biochem..

[8] Wu F., Li Q., Yan H., Zhang D., Jiang G., Jiang Y., Duan X. (2016). Characteristics of Three Thioredoxin Genes and Their Role in Chilling Tolerance of Harvested Banana Fruit. Int. J. Mol. Sci..

[9] Svensson M.J., Larsson J. (2007). Thioredoxin-2 affects lifespan and oxidative stress in Drosophila. Hereditas.

[10] Ogata F.T., Batista W.L., Sartori A., Gesteira T.F., Masutani H., Arai R.J., Yodoi J., Stern A., Monteiro H.P. (2013). Nitrosative/oxidative stress conditions regulate thioredoxin-interacting protein (TXNIP) expression and thioredoxin-1 (TRX-1) nuclear localization. PLoS ONE.

[11] Sugano E., Murayama N., Takahashi M., Tabata K., Tamai M., Tomita H. (2013). Essential role of thioredoxin 2 in mitigating oxidative stress in retinal epithelial cells. J. Ophthalmol..

[12] Chibani K., Pucker B., Dietz K.J., Cavanagh A. (2021). Genome-wide analysis and transcriptional regulation of the typical and atypical thioredoxins in Arabidopsis thaliana. FEBS Lett..

[13] Montrichard F., Alkhalfioui F., Yano H., Vensel W.H., Hurkman W.J., Buchanan B.B. (2009). Thioredoxin targets in plants: The first 30 years. J. Proteom..

[14] Sainz M.M., Filippi C.V., Eastman G., Sotelo-Silveira J., Borsani O., Sotelo-Silveira M. (2022). Analysis of Thioredoxins and Glutaredoxins in Soybean: Evidence of Translational Regulation under Water Restriction. Antioxidants.

[15] Liu H., Li Y., Huang X. (2021). Genome-Wide Analysis of the Thioredoxin Gene Family in Gossypium hirsutum L. and the Role of the Atypical Thioredoxin Gene GhTRXL3-2 in Flowering. J. Plant Biol..

[16] Meyer Y., Siala W., Bashandy T., Riondet C., Vignols F., Reichheld J.P. (2008). Glutaredoxins and thioredoxins in plants. Biochim. Biophys. Acta (BBA)—Mol. Cell Res..

[17] Tada Y., Spoel S.H., Pajerowska-Mukhtar K., Mou Z., Song J., Wang C., Zuo J., Dong X. (2008). Plant immunity requires conformational changes [corrected] of NPR1 via S-nitrosylation and thioredoxins. Science.

[18] Kneeshaw S., Gelineau S., Tada Y., Loake G.J., Spoel S.H. (2014). Selective protein denitrosylation activity of Thioredoxin-h5 modulates plant Immunity. Mol. Cell.

[19] Lee E.S., Park J.H., Wi S.D., Chae H.B., Paeng S.K., Bae S.B., Phan K.A.T., Lee S.Y. (2021). Arabidopsis Disulfide Reductase, Trx-h2, Functions as an RNA Chaperone under Cold Stress. Appl. Sci..

[20] Zhai J., Qi Q., Wang M., Yan J., Li K., Xu H. (2022). Overexpression of tomato thioredoxin h (SlTrxh) enhances excess nitrate stress tolerance in transgenic tobacco interacting with SlPrx protein. Plant Sci..

[21] Chung Y.B., Lee H., Hwang S., Seo H.Y., Suh H.J., Jo K. (2021). Effect of capsaicinoids in hot pepper powder on microbial community and free sugar during kimchi fermentation. J. Food Sci..

[22] Liu T., Wan Y., Meng Y., Zhou Q., Li B., Chen Y., Wang L. (2023). Capsaicin: A Novel Approach to the Treatment of Functional Dyspepsia. Mol. Nutr. Food Res..

[23] Tiamiyu Q.O., Adebayo S.E., Ibrahim N. (2023). Recent advances on postharvest technologies of bell pepper: A review. Heliyon.

[24] Wang B., Dai T., Sun W., Wei Y., Ren J., Zhang L., Zhang M., Zhou F. (2021). Protein N-myristoylation: Functions and mechanisms in control of innate immunity. Cell. Mol. Immunol..

[25] Majeran W., Le Caer J.P., Ponnala L., Meinnel T., Giglione C. (2018). Targeted Profiling of Arabidopsis thaliana Subproteomes Illuminates Co- and Posttranslationally N-Terminal Myristoylated Proteins. Plant Cell.

[26] Traverso J.A., Micalella C., Martinez A., Brown S.C., Satiat-Jeunemaître B., Meinnel T., Giglione C. (2013). Roles of N-terminal fatty acid acylations in membrane compartment partitioning: Arabidopsis h-type thioredoxins as a case study. Plant Cell.

[27] Muhammad Aslam M., Waseem M., Jakada B.H., Okal E.J., Lei Z., Saqib H.S.A., Yuan W., Xu W., Zhang Q. (2022). Mechanisms of Abscisic Acid-Mediated Drought Stress Responses in Plants. Int. J. Mol. Sci..

[28] Rehman A., Azhar M.T., Hinze L., Qayyum A., Li H., Peng Z., Qin G., Jia Y., Pan Z., He S. (2021). Insight into abscisic acid perception and signaling to increase plant tolerance to abiotic stress. J. Plant Interact..

[29] Marcotte W.R., Russell S.H., Quatrano R.S. (1989). Abscisic acid-responsive sequences from the em gene of wheat. Plant Cell.

[30] Guiltinan M.J., Marcotte W.R., Quatrano R.S. (1990). A plant leucine zipper protein that recognizes an abscisic acid response element. Science.

[31] Agarwal M., Hao Y., Kapoor A., Dong C.H., Fujii H., Zheng X., Zhu J.K. (2006). A R2R3 type MYB transcription factor is involved in the cold regulation of CBF genes and in acquired freezing tolerance. J. Biol. Chem..

[32] Zhou M.Q., Shen C., Wu L.H., Tang K.X., Lin J. (2011). CBF-dependent signaling pathway: A key responder to low temperature stress in plants. Crit. Rev. Biotechnol..

[33] Duan X., Wang Z., Zhang Y., Li H., Yang M., Yin H., Cui J., Chai H., Gao Y., Hu G. (2022). Overexpression of a Thioredoxin-Protein-Encoding Gene, MsTRX, from Medicago sativa Enhances Salt Tolerance to Transgenic Tobacco. Agronomy.

[34] Park J.H., Lee E.S., Chae H.B., Paeng S.K., Wi S.D., Bae S.B., Thi Phan K.A., Lee S.Y. (2021). Disulfide reductase activity of thioredoxin-h2 imparts cold tolerance in Arabidopsis. Biochem. Biophys. Res. Commun..

[35] Meyer Y., Reichheld J.P., Vignols F. (2005). Thioredoxins in Arabidopsis and other plants. Photosynth. Res..

[36] Yoshida K., Hara S., Hisabori T. (2015). Thioredoxin Selectivity for Thiol-based Redox Regulation of Target Proteins in Chloroplasts. J. Biol. Chem..

[37] Thormählen I., Zupok A., Rescher J., Leger J., Weissenberger S., Groysman J., Orwat A., Chatel-Innocenti G., Issakidis-Bourguet E., Armbruster U. (2017). Thioredoxins Play a Crucial Role in Dynamic Acclimation of Photosynthesis in Fluctuating Light. Mol. Plant.

[38] Ying Y., Yue W., Wang S., Li S., Wang M., Zhao Y., Wang C., Mao C., Whelan J., Shou H. (2017). Two h-Type Thioredoxins Interact with the E2 Ubiquitin Conjugase PHO2 to Fine-Tune Phosphate Homeostasis in Rice. Plant Physiol..

[39] Cejudo F.J., González M.C., Pérez-Ruiz J.M. (2021). Redox regulation of chloroplast metabolism. Plant Physiol..

[40] Meng L., Wong J.H., Feldman L.J., Lemaux P.G., Buchanan B.B. (2010). A membrane-associated thioredoxin required for plant growth moves from cell to cell, suggestive of a role in intercellular communication. Proc. Natl. Acad. Sci. USA.

[41] Lee E.S., Park J.H., Wi S.D., Kang C.H., Chi Y.H., Chae H.B., Paeng S.K., Ji M.G., Kim W.Y., Kim M.G. (2021). Redox-dependent structural switch and CBF activation confer freezing tolerance in plants. Nat. Plants.

[42] Yoshida K., Hisabori T. (2023). Current Insights into the Redox Regulation Network in Plant Chloroplasts. Plant Cell Physiol..

[43] Yano H. (2014). Ongoing applicative studies of plant thioredoxins. Mol. Plant.

[44] Zhang L., Liu C., Cheng F.F., Guo X.Y., Li Y.X., Wang A.Y., Zhu J.B. (2021). Molecular cloning and functional analysis of the thioredoxin gene SikTrxh from Saussurea involucrata. Biol. Plant..

[45] Park S.K., Jung Y.J., Lee J.R., Lee Y.M., Jang H.H., Lee S.S., Park J.H., Kim S.Y., Moon J.C., Lee S.Y. (2009). Heat-shock and redox-dependent functional switching of an h-type Arabidopsis thioredoxin from a disulfide reductase to a molecular chaperone. Plant Physiol..

[46] Arnaiz A., Romero-Puertas M.C., Santamaria M.E., Rosa-Diaz I., Arbona V., Munoz A., Grbic V., Gonzalez-Melendi P., Mar Castellano M., Sandalio L.M. (2023). The Arabidopsis thioredoxin TRXh5regulates the S-nitrosylation pattern of the TIRK receptor being both proteins essential in the modulation of defences to Tetranychus urticae. Redox Biol..

[47] Kinkema M., Fan W., Dong X. (2000). Nuclear localization of NPR1 is required for activation of PR gene expression. Plant Cell.

[48] Kumar S., Stecher G., Li M., Knyaz C., Tamura K. (2018). MEGA X: Molecular Evolutionary Genetics Analysis across Computing Platforms. Mol. Biol. Evol..

[49] Offenborn J.N., Waadt R., Kudla J. (2015). Visualization and translocation of ternary Calcineurin-A/Calcineurin-B/Calmodulin-2 protein complexes by dual-color trimolecular fluorescence complementation. New Phytol..

[50] Huh S.U. (2022). Optimization of immune receptor-related hypersensitive cell death response assay using agrobacterium-mediated transient expression in tobacco plants. Plant Methods.

[51] Lescot M., Dehais P., Thijs G., Marchal K., Moreau Y., Van de Peer Y., Rouze P., Rombauts S. (2002). PlantCARE, a database of plant cis-acting regulatory elements and a portal to tools for in silico analysis of promoter sequences. Nucleic Acids Res..

