# Supplementary material for: Members of the Capsicum annuum CaTrxh Family Respond to High Temperature and Exhibit Dynamic Hetero/Homo Interactions
Source: Int J Mol Sci. 2024 Jan 31;25(3):1729. doi: 10.3390/ijms25031729 (PMC10855718; doi:10.3390/ijms25031729)

Supplemental Figures (Figure S1-S4)

Supplemental Figure S1

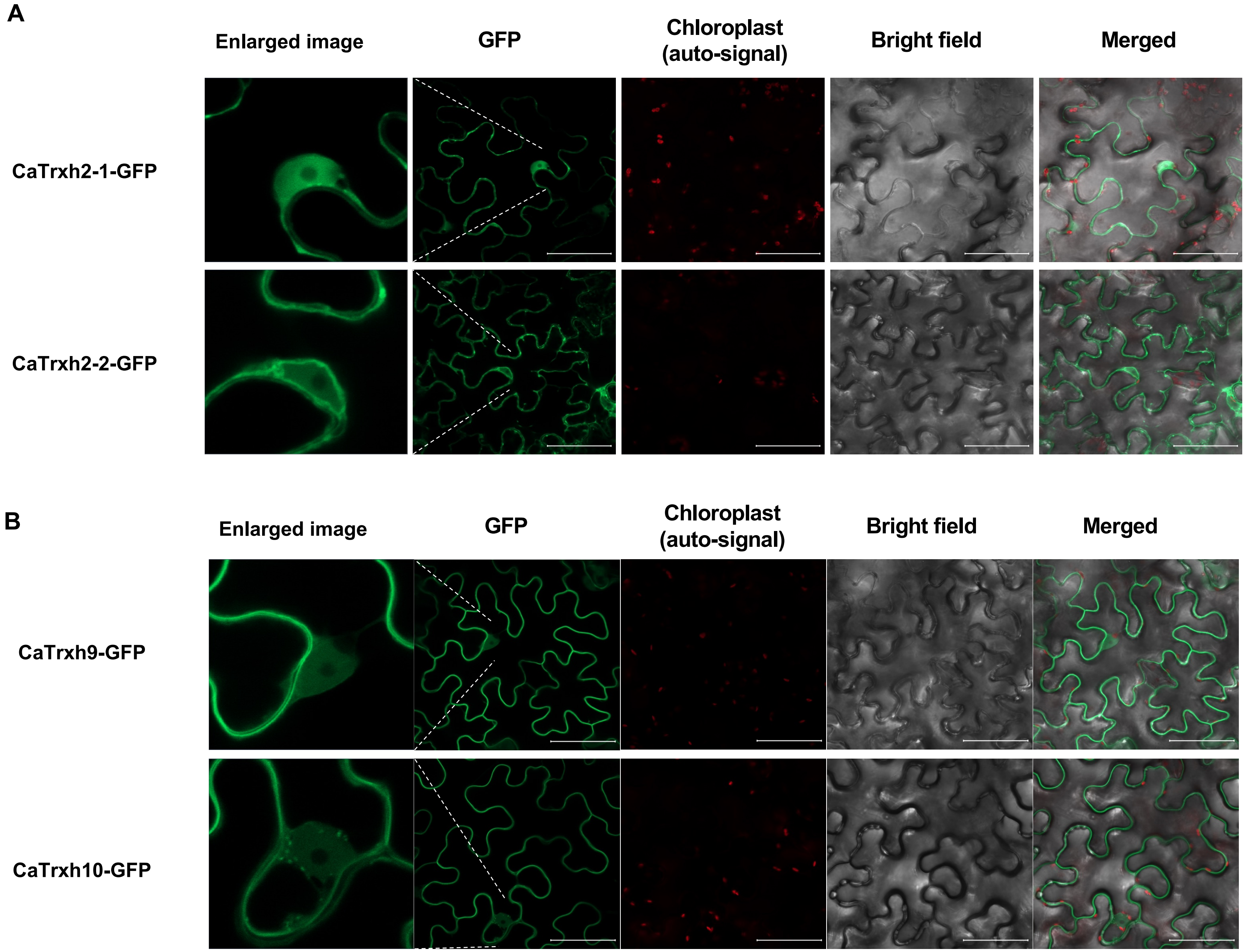

Supplemental Figure S2

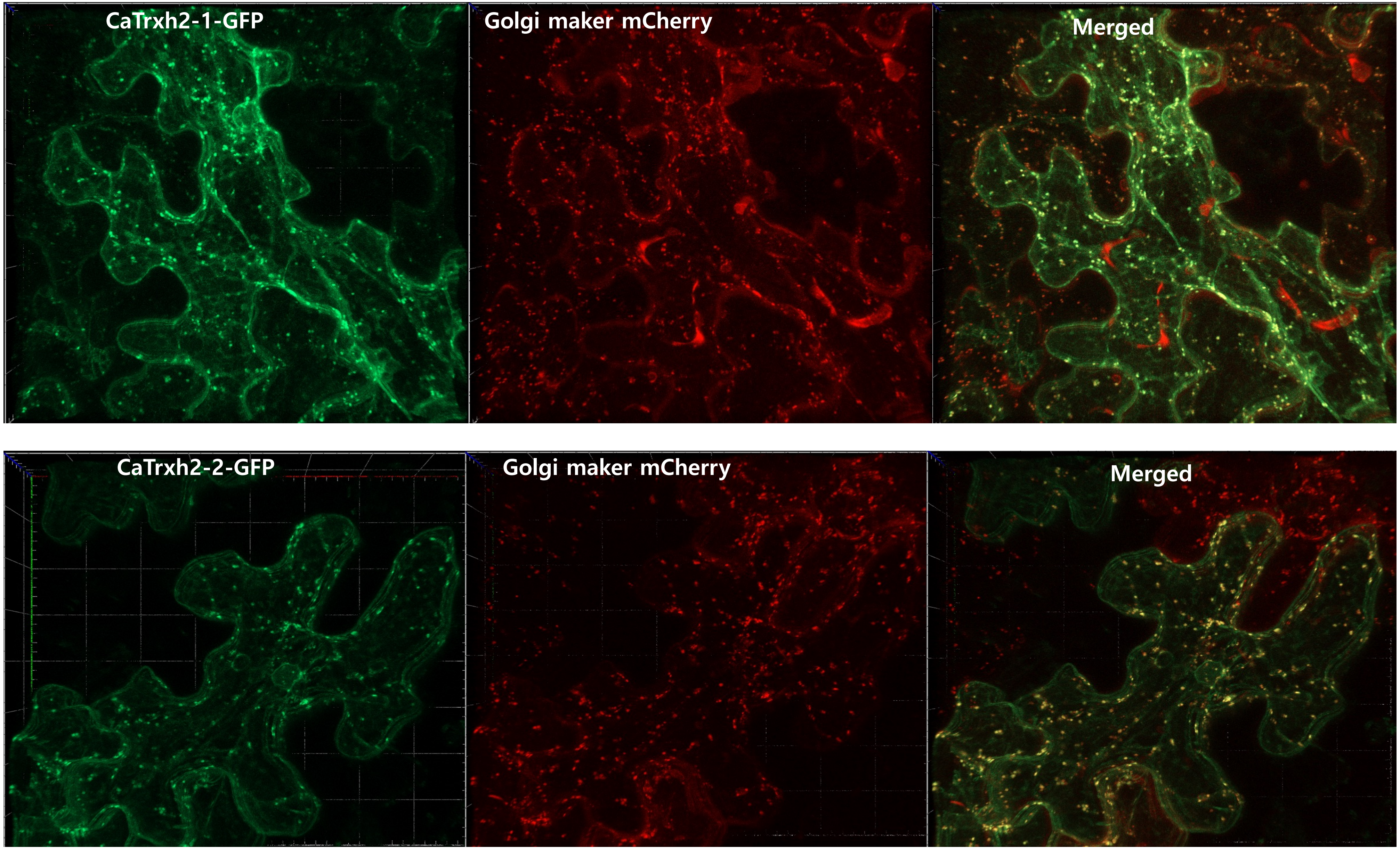

Supplemental Figure S3

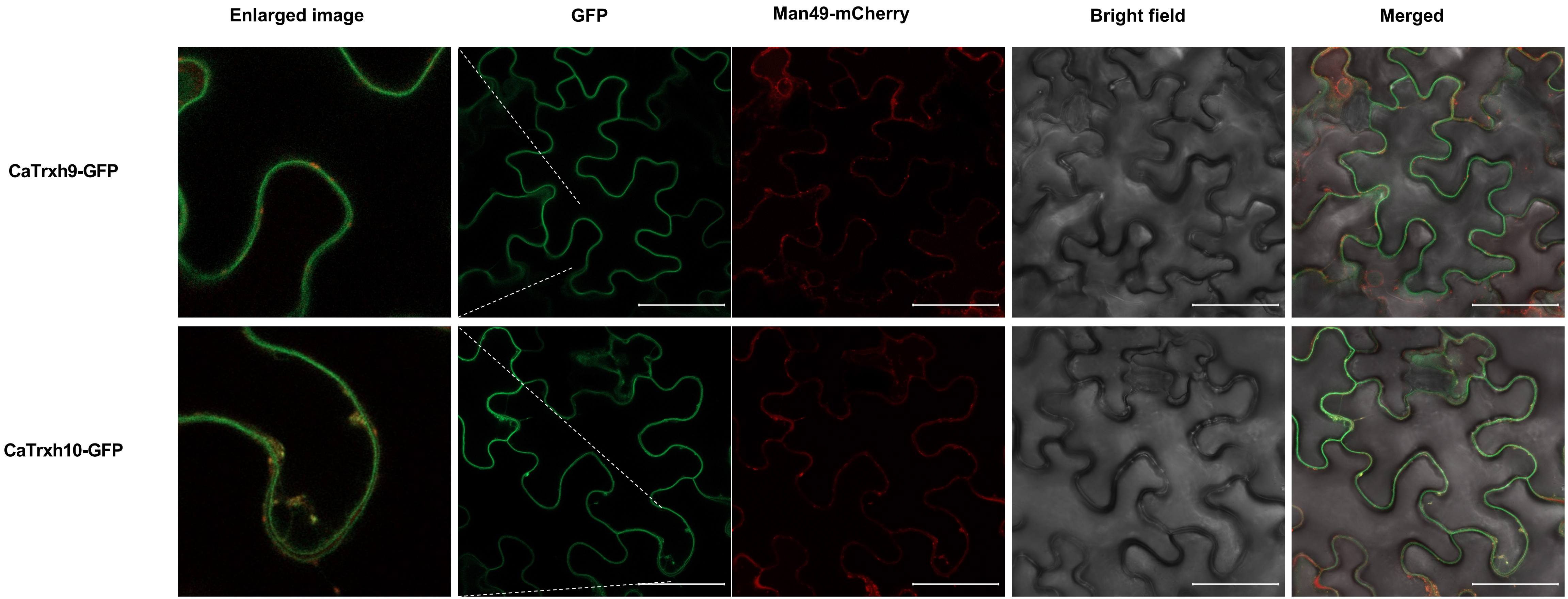

Supplemental Figure S4

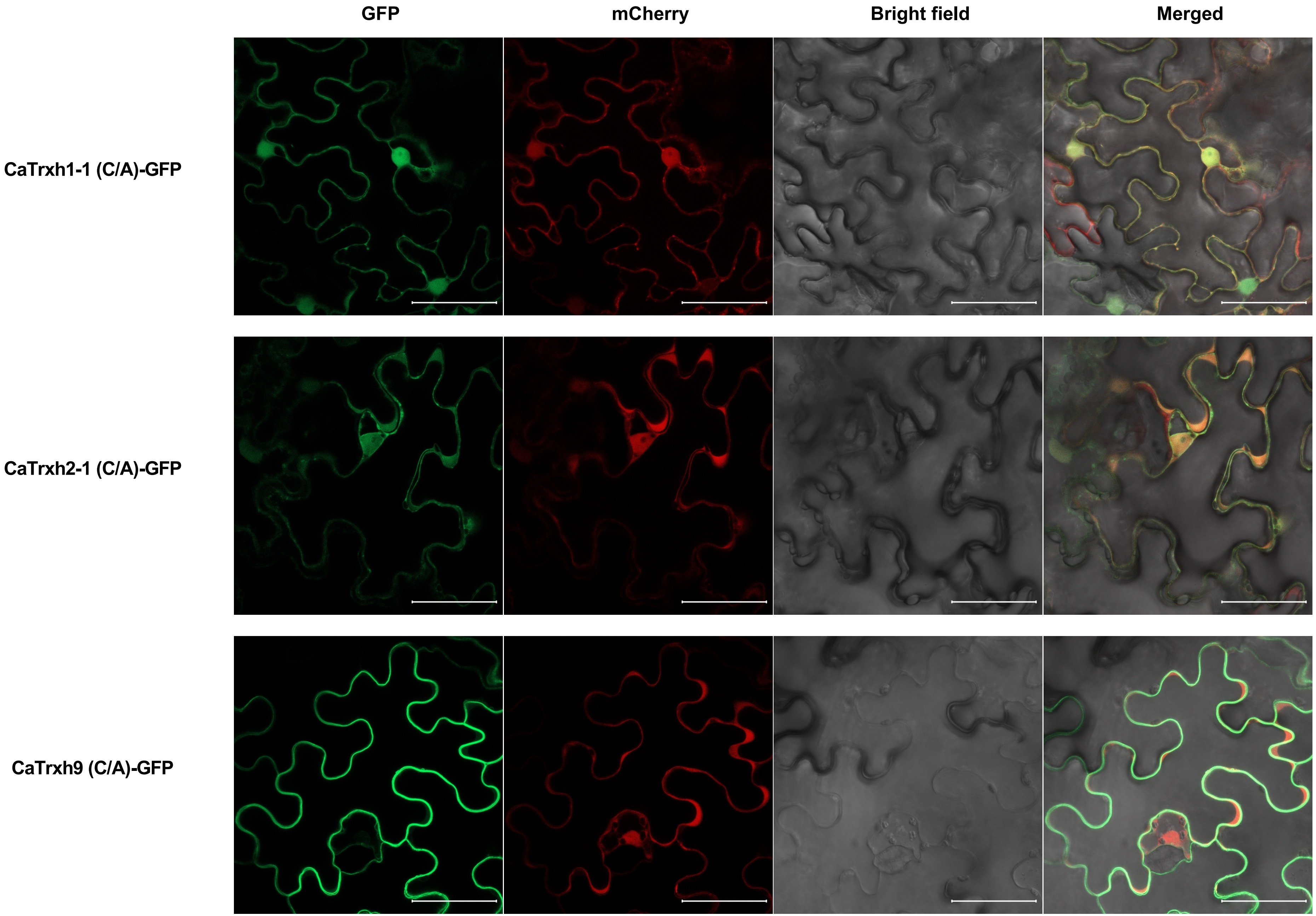

Supplement: Supplementary file 1 [file ijms-25-01729-s001.zip › 2024_CaTrxh_Hong_supplemental FigureS1-S4_revision.pdf]
