# Supplementary material for: Members of the Capsicum annuum CaTrxh Family Respond to High Temperature and Exhibit Dynamic Hetero/Homo Interactions
Source: Int J Mol Sci. 2024 Jan 31;25(3):1729. doi: 10.3390/ijms25031729 (PMC10855718; doi:10.3390/ijms25031729)
Supplement: Supplementary file 1 [file ijms-25-01729-s001.zip › Table S1. RT-PCR, qRT-PCR, and cloning primers in this study.pdf]

Table S1. RT-PCR, qRT-PCR, and cloning primers in this study

Primers	Forward Primer sequence (5' to 3')	Reverse Primer sequence (5' to 3')	Purpose
CaTrxh1-1	GGATCCATGGCTGCTACTTCATCTGA	GGATCCTTGAGGAATATGACATGGGG	RT-PCR/qRT-PCR
CaTrxh1-2	GGATCCATGGCAGAGGAAGGGCAAGT	GGATCCTCCACATCCACTTTCAGGAA	
CaTrxh1-3	GGATCCGATAGTCGTGGACTTCACTG	GGATCCCCTTATCGACGATATTCCCC	
CaTrxh2-1	GGATCCATGGGTGCTAACTACTCAGC	GGATCCACTGGATCCATGTATTTGCA	
CaTrxh2-2	GGATCCATGGGAAGCTTCCTTTCAAG	GGATCCAACTTGCAAGGGCCACACCA	
CaTrxh9	GGATCCAGACAAAAGGAGAAACACGT	GGATCCACTGGATCCATGTATTTGCA	
CaTrxh10	GGATCCGCAAGATTGCGGTGGTAAAT	GGATCCTTCGCCGCCTACTAGTTTGT	
CaActin	GACGTGACCTAACTGATAACCTGAT	CTCTCAGCACCAATGGTAATAACTT	
CaTrxh1-1	CTCGAGATGGCTGCTACTTCATCTGA	CCCGGGAGCTGTCACAGTAGCAGGAG	Cloning
CaTrxh1-2	CTCGAGATGGCAGAGGAAGGGCAAGT	CCCGGGGGCTGATGAGGAGCTACTAC	
CaTrxh1-3	CTCGAGATGGCAGAAGAGGGACAAGT	CCCGGGGGCTGATGCACTACCAATGT	
CaTrxh2-1	CTCGAGATGGGTGCTAACTACTCAGC	CCCGGGGATGAACACAGCTCTGTGTT	
CaTrxh2-2	CTCGAGATGGGAAGCTTCCTTTCAAG	CCCGGGGGCAGCGTATTTAGGGGCTT	
CaTrxh9	CTCGAGATGGGGATCTCTGATACGGT	CCCGGGCTGCGGTTGTTTCTCGCATG	
CaTrxh10	GGATCCATGGGGCACTGCTGGACTAA	CTCGAGTCCCGGGGACCTTGTAGACT	
SAUR63	GGATCCATGATAAACGCAAAGAAGC	CCCGGGAAATACAAGCAACTGTTGCG	
CaTrxh1-1	CCTGCACGTTTTATTGCCCCAATTC	ACCTGCCCAGGATGCGGTAAAATC	Mutagenesis
CaTrxh2-1	CCTGCTAAATACATGGATCCAGTTC	ACCAGCCCATGTAGCTGTGAAGTC	
CaTrxh9	CCAGCTAGAATGATTGCTCCATTC	ACCAGCCCATGAAGCACTGAAATTTG	
